# Continuous Flow Synthesis of Heterocycles: A Recent Update on the Flow Synthesis of Indoles

**DOI:** 10.3390/molecules25143242

**Published:** 2020-07-16

**Authors:** Marco Colella, Leonardo Degennaro, Renzo Luisi

**Affiliations:** Flow Chemistry and Microreactor Technology FLAME-Lab, Department of Pharmacy—Drug Sciences, University of Bari “A. Moro” Via E. Orabona 4, 70125 Bari, Italy; marco.colella1992@gmail.com

**Keywords:** flow chemistry, indoles, organic synthesis

## Abstract

Indole derivatives are among the most useful and interesting heterocycles employed in drug discovery and medicinal chemistry. In addition, flow chemistry and flow technology are changing the synthetic paradigm in the field of modern synthesis. In this review, the role of flow technology in the preparation of indole derivatives is showcased. Selected examples have been described with the aim to provide readers with an overview on the tactics and technologies used for targeting indole scaffolds.

## 1. Introduction

**Scheme 1:**
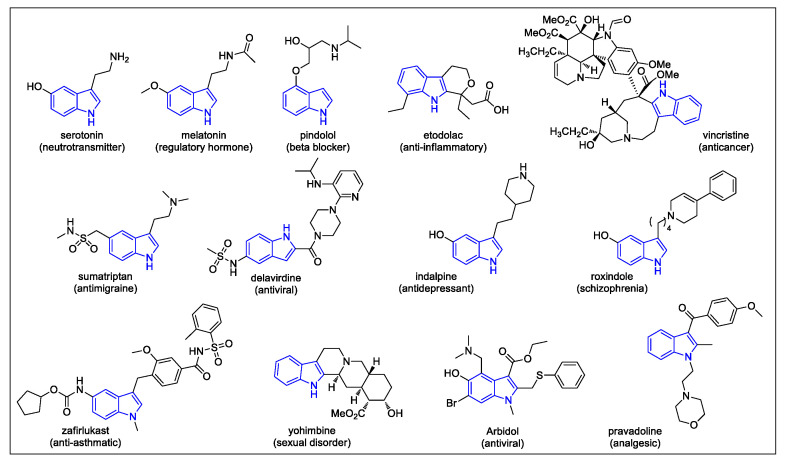
Selected examples of indole-containing biologically active compounds.

Among heterocycles, the indole ring is one of the most ubiquitous in nature as well as in man-made products, such as pharmaceuticals, agrochemicals, dyes, and materials [1,2]. In particular, the indole core appears as a substructure of various biologically active compounds, including the neurotransmitter serotonin; the regulatory hormone melatonin; and various medicines, such as antihypertensive, anti-inflammatory, antimigraine, anti-tumor, and many others (Scheme 1) [3]. In addition, indole is a privileged scaffold in drug discovery programs targeting new antivirals. Examples of marketed indole-containing antiviral drugs include Arbidol and Delavirdine. Arbidol is used for the treatment and prophylactic prevention of influenza A and B virus and SARS. Delavirdine is a first-generation non-nucleoside reverse transcriptase inhibitor that was approved by the FDA in 1997 for the treatment of human immunodeficiency virus type 1 (HIV-1) [4,5].

Since its first isolation by Baeyer from the reaction of indigo with oleum (indole = indigo + oleum) [6], many routes have been developed for the indole nucleus preparation, although the importance of this nucleus still impels a never-ending quest to design alternative approaches that are cost-effective, highly productive, and sustainable. For this purpose, not only are “new routes for a classic target” [1] highly demanded, but also the implementation of currently available methods taking advantage of modern technology tools is desired. In pursuing this objective, flow microreactor technology represents a unique resource due to its widely recognized potential in the intensification of synthetic processes. Indeed, the introduction of flow microreactors has paved the way to a breakthrough in different areas of organic synthesis thanks to different advantages over traditional batch processes, such as large surface-to-volume ratio, efficient mass and heat transfer, precise and fast mixing, intrinsic safety, lower solvent and reagent consumption, easier scalability, higher productivity, and reduced footprint and capital investment [7,8,9,10,11]. Moreover, in recent years flow microreactor technology has not only allowed the refinement of various synthetic methodologies but has also disclosed processes which are difficult to perform under batch conditions [12,13]. In this review, recent flow chemistry approaches developed for strengthening the tactics of indole ring preparation and functionalization will be described.

## 2. Synthesis of Indole Ring under Continuous Flow Conditions

### 2.1. Fischer Indole Synthesis

**Scheme 2:**
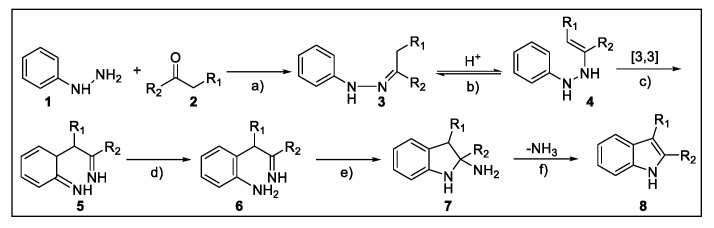
Key steps of the proposed mechanism for the Fischer indolization: (**a**) *N*-arylhydrazone generation, (**b**) enamine formation, (**c**) [3,3]-sigmatropic rearrangement, (**d**) re-aromatization, (**e**) cyclization, (**f**) indole formation.

The Fischer approach, proposed for the first time in 1883 [14], is one of the oldest reactions in organic chemistry [15] but is still the pillar for the preparation of indoles [16]. In the presence of the appropriate acid or acid catalyst, the desired indole is typically obtained by heating ketone **2** (or aldehyde) and arylhydrazine **1** without the need for the isolation of the enolizable *N*-arylhydrazone intermediate **3**. Mechanistically (Scheme 2), it is likely that the tautomer **4** undergoes a [3,3]-sigmatropic rearrangement, giving the bis iminobenzylketone **5** with the loss of aromaticity [16,17,18]. The latter furnishes the desired indole **8** as a result of rearomatization, cyclization to cyclic aminal 7, and the formation of an indole ring with the loss of ammonia. 

The possibility to transfer this transformation in a continuous flow system has attracted different research teams, among which are the groups of Bagley [19] and Kappe [20], whose seminal works demonstrated some important advantages related to the flow technology with respect to the traditional methods. In particular, the adoption of microwave heating or the use of high-temperature/high-pressure flow systems allowed them to improve reaction yields, limiting side-product formation, and to reduce the reaction time, making this process not only suitable for continuous flow but also superior in terms of productivity with respect to the batch. In the following two sections, various contributions to this field will be presented, highlighting the main aspects and differences between the flow microwave-assisted reactions and conventional heating transformations.

#### 2.1.1. Continuous Flow Microwave-Assisted Fischer Indolization

**Scheme 3:**
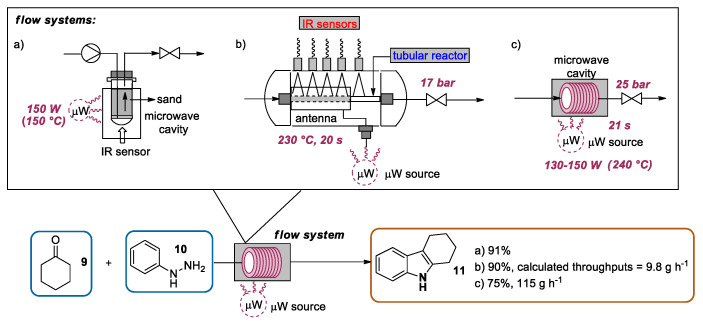
CF-MAOS approach applied to Fischer indole synthesis: (**a**) first example proposed by Bagley; (**b**) nonresonant microwave system for continuous flow synthesis developed by Larhed; (**c**) bench-top single-mode microwave setup presented by Akai.

From the early experiments conducted in domestic ovens in the 1980s [21,22] to the use of safe and advanced equipment specifically designed for organic reactions [19,23,24,25,26,27], microwave-assisted organic synthesis (MAOS) has proved to be a convenient alternative to conventional heating. Typically, the use of microwave energy ensures a rapid dielectric heating of the reaction mixture to temperatures above the boiling point of the solvent [28,29], thus enabling an increase in reaction rates [30,31]; the fast optimization of reaction procedures [19]; and, in many cases, higher yields [32,33]. Under microwave irradiation, the heating profile is uniform and highly controlled, leading to more reproducible reactions [25]. Moreover, in some cases the use of microwave heating resulted in higher chemo-, regio-, or stereoselectivities as a result of the different absorption and transmission of the energy with respect to conventional heating [34]. However, scaling up microwave-assisted organic reactions is limited by the penetration depth of microwaves (few centimeters), confining this approach to small-scale MW devices [23]. Although the combined use of large multimode MW systems [35,36] and stop-flow synthesis [37] has partially circumvented these limitations, alternative approaches to microwave-mediated large-scale synthesis are highly demanded [38]. In this context, a combination of CF and microwave-assisted organic synthesis (CF-MAOS) was originally proposed by Strauss and coworkers in 1990s [39,40]. In the following years, different groups have applied the CF-MAOS approach also to Fischer synthesis for the preparation of indole scaffolds. The first example was reported by Bagley et al., who designed a CF microwave reactor consisting of a standard pressure-rated glass tube (10 mL) filled with sand (ca. 12 g), which was introduced into the cavity of a self-tunable monomodal microwave synthesizer [19]. The tuning of the microwave power enabled the regulation of the reaction temperature, which was directly measured using an IR sensor. A solution of cyclohexanone **9** and phenylhydrazine **10** in glacial AcOH was introduced into the flow cell at a flow rate of 0.5 mL min^−1^ by using an HPLC pump, and the reaction mixture was heated to 150 °C using a microwave power of 150 W. The combined solutions were evaporated, furnishing, after work-up and purification by recrystallization, the indole **11** in a 91% yield (Scheme 3a).

In the same field, Larhed and coworkers designed a nonresonant microwave apparatus for organic synthesis, consisting of a helical microwave antenna of Al-Cu material wrapped around a tubular reactor made of microwave-transparent borosilicate glass [23]. The Fischer indole synthesis was selected, along with other transformations, to test the usefulness of this setup. Hence, a solution of cyclohexanone **9** and a solution of phenylhydrazine **10** were pumped through the CF-MAOS system by a Syrris Asia syringe pump. After the optimization study, using 230 °C as the temperature and 20 s as the residence time, indole **11** was obtained in a 90% yield with a noteworthy estimated throughput of 9.8 g·h^−1^ (57.2 mmol·h^−1^) (Scheme 3b). Some years later, Akai and coworkers developed a bench-top single-mode microwave apparatus for continuous flow organic synthesis [38]. In this case, the Fischer indolization was selected as a representative transformation to evaluate the apparatus performance. Hence, a solution of cyclohexanone **9** and a solution of phenylhydrazine **10** were separately pumped at the same flow rate through a borosilicate helical glass tube reactor placed into the resonant cavity under a pressure of 25 bar. The optimization study was conducted by changing the solution flow rates (13–17 mL·min^−1^), the irradiation power (130–190 W), and the solution concentrations and evaluating the GC yield of product **11**. A reaction scale-up was conducted under optimized conditions. In particular, a 2.0 M solution of cyclohexanone **9** in AcOH and a 2.2 M solution of phenylhydrazine **10** in MeCN were flowed into the system using a total flow rate of 15 mL·min^−1^ (corresponding to a residence time of 21 s) and using an irradiation power of 145 W. Under steady state conditions at 240 °C, 115 g (75%) of product **11** were collected within 60 min. During the collection, to avoid solvent boiling the exit temperature was kept constant by tuning the irradiation power between 130 and 150 W (Scheme 3c).

**Scheme 4:**
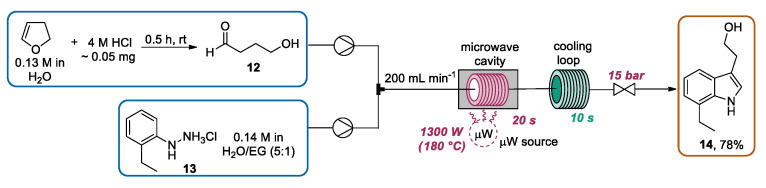
Fischer indole synthesis of 7-ET under microwave-assisted continuous flow conditions.

In recent years, different works focused on the continuous flow Fischer synthesis for the preparation of tryptophol and derivatives, which have been recognized as key intermediates for the construction of relevant bioactive compounds, such as some antimigraine triptans [41,42,43,44,45] and indoramine, a selective alpha-1 adrenergic receptor antagonist. In particular, in 2017 Yu and Lv [46] reported the application of CF-MAOS methodology for the preparation of 7-ethyltryptophol (7-ET) **14**, a relevant intermediate en route to the potent non-steroidal anti-inflammatory and analgesic etodolac [47,48,49]. In this work, using a total flow rate of 200 mL·min^−1^, a solution of 4-hydroxybutanal **12** (prepared by adding a drop of 4 M hydrochloric acid to a solution of 2,3-dihydrofuran) in H_2_O and a solution of 2-ethylphenylhydrazine hydrochloride **13** in water/ethylene glycol (EG) preheated at 80 °C were pumped through the microwave-heating residence loop with a residence time of 20 s. Using these conditions, an exit temperature of 180 °C was achieved. Then, the mixture flowed through a cooling loop with a residence time of 10 s and was diluted with water and extracted with methyl tert-butyl ether (MTBE). After the evaporation of the organic phase, 7-ET was obtained in a 78% isolated yield. In this contribution, the microwave heating enabled the use of a very short residence time (30 s), inhibiting the formation of side-products derived from the reaction of 7-ET with aldehyde excess (Scheme 4).

**Scheme 5:**
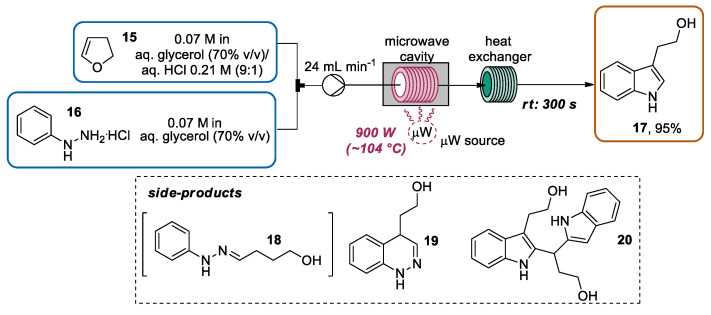
FC-MAOS setup for the preparation of tryptophol.

In the same year, Gaikar and co-workers developed a Fischer indolization for the synthesis of tryptophol **17**, starting from 2,3-DHF **15** and phenyl hydrazine hydrochloride (PHH) **16** by using the CF-MAOS approach [50]. The initial experiments, performed using conventional oil bath heating, showed that raising the reaction temperature and the flow rates resulted in a higher conversion of PHH **16** and higher yields of tryptophol **17**. However, at higher temperatures two side products—a cinnoline derivative **19** obtained by a different cyclization reaction of the intermediate hydrazine **18**, and a bis-indole **20**, a dimer formed by a consecutive reaction between **17** and one more molecule of **15**—were detected. A kinetic model for this reaction scenario was proposed using experimental data and a COMSOL simulation, determining the activation energies for the generation of derivatives **18** (36.8 kJ/mol), **17** (40.1 kJ/mol), **20** (46.9 kJ/mol), and **19** (67.7 kJ/mol). Hence, while the activation energy for the generation of **18** was low, a higher temperature was necessary to pass the energy barrier of tryptophol **17** formation. On the other hand, an excessive amount of energy promoted the formation of side products. Hence, a microwave heating approach was considered. The efficiency of the microwave heating transfer allowed the researchers to provide the required energy for the effective cyclization of hydrazine **18** to tryptophol in a shorter time, limiting the formation of by-products. In particular, under optimized conditions using a peristaltic pump, a solution of **15** and a solution of **16** (PHH) in environmentally friendly aqueous glycerol solution were separately introduced through a quartz reactor tube placed in the microwave cavity with a residence time of 300 s. The power input was set to 900 W, furnishing a temperature of about 104 °C at the exit of the reactor. Using a heat exchanger, the mixture was cooled, collected, and after work-up furnished the desired tryptophol **17** in a 95% yield (Scheme 5).

#### 2.1.2. Continuous Flow Fischer Indole Synthesis Mediated by Conventional Heating

**Scheme 6:**
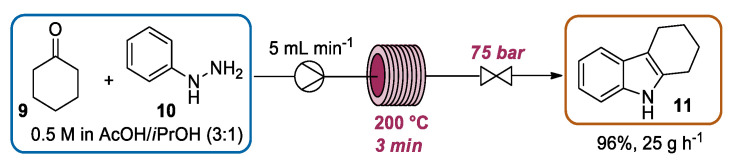
Continuous flow Fischer indole synthesis under high-temperature/pressure conditions.

As described in the previous section, microwave technology enables a significant improvement of a chemical process through the heating of the reaction mixture to temperatures that exceed the boiling point of the solvent. In 2009, Kappe presented a seminal work in which the appealing possibility to exploit a heated flow system for the indole synthesis was demonstrated [20]. For this study, a high-temperature/pressure flow reactor embedded with HPLC pumps to introduce the reactant solution, a stainless steel coil heated by an electric heat exchanger (up to 350 °C), thermocouples to measure the temperature at the exit of the coil, a system pressure sensor, and a system pressure valve to set the pressure value were employed (Scheme 6). The use of a high pressure (up to 200 bar) allowed researchers to exploit lower boiling solvents in or near their supercritical state. The authors selected the Fischer indole synthesis as one of the model reactions for testing the flow system. In particular, inspired by Bagley’s work, a mixture of cyclohexanone **9** and phenylhydrazine **10** in acetic acid/iso-propanol (3:1) was introduced with a flow rate of 5 mL min^−1^ into a 16 mL stainless steel coil heated to 200 °C at 75 bar. With a residence time of ca. 3 min, the indole **11** was obtained in 96% yield with productivity of 25 g·h^−1^. The reduced reaction time under the high-temperature/pressure conditions in combination with the intrinsic easy scalability of flow chemistry makes this approach particularly attractive in terms of productivity with respect to conventional batch procedures. 

**Scheme 7:**
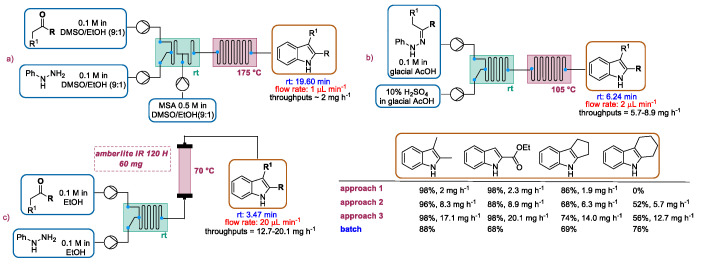
Three different approaches for the continuous flow Fischer synthesis of a series of indoles: (**a**) initial low-productivity set-up; (**b**,**c**) modified versions of the system enabling lower back-pressure and higher productivity.

In 2010, Watts and co-workers reported the Fischer synthesis of a series of indoles using three different continuous flow approaches which were compared, especially in terms of productivity [51]. In this report, the authors described the first flow reactor, depicted in Scheme 7, which was able to merge the solutions of ketones and phenylhydrazine (in DMSO with 10% of ethanol) into a glass microreactor by syringe pumps generating the phenylhydrazone intermediates. The resulting solution was subsequently mixed with a solution of MSA (methanesulfonic acid), which upon heating promoted the cyclization of phenylhydrazones via a [3,3]-sigmatropic rearrangement, giving the substituted indoles. Despite the high conversion for some ketones, the throughput (around 2 mgh^−1^) was not satisfactory, likely due to the high system backpressure. For this reason, the authors proposed a second approach (Scheme 7b) in which glacial acetic acid and sulfuric acid were respectively selected as the solvent and catalyst. Using these conditions, cyclization occurred faster, allowing the researchers to reduce the length of the reactor and consequently the backpressure of the system. A productivity of 5.7–8.9 mg·h^−1^ was achieved. The authors proposed a third methodology based on heterogeneous catalyzed Fischer indole synthesis (Scheme 7c). Namely, phenylhydrazones, generated by mixing ketones and phenylhydrazine in ethanol in a T-chip, were cyclized in a capillary packed with Amberlite IR 120H at 70 °C. The low backpressure generated by this setup allowed a significant increase in flow rates, achieving an acid-free throughput of 12.7–20.1 mg·h^−1^. 

**Scheme 8:**
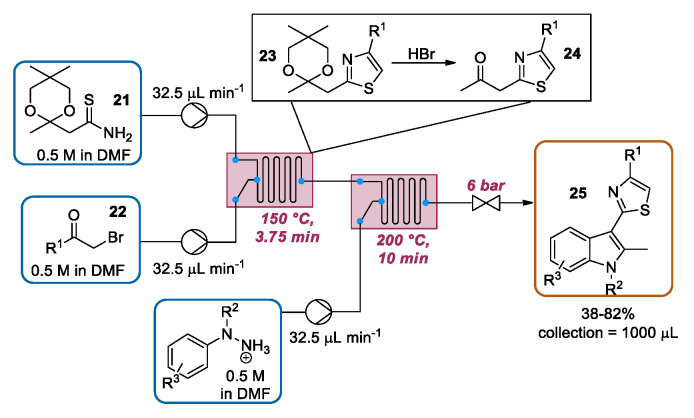
Multistep continuous flow synthesis of various indolylthiazoles.

In 2012, Cosford and co-workers presented an uninterrupted multistep continuous flow synthesis of 2-(1*H*-indol-3-yl)thiazoles in a Syrris AFRICA^®^ station, consisting of a sequential Hantzsch thiazole synthesis, deketalization, and Fischer indole synthesis. The ketal-protected thioamide **21** was reacted with α-bromoketone **22** in a glass reactor heated to 150 °C, generating ketal thiazole **23**. The latter was directly deprotected with HBr, generated during thiazole ring formation, giving the desired ketothiazole **24**, which was then mixed with hydrazine hydrochloride **16** in a heated (200 °C) glass reactor, furnishing indolylthiazole **25**. The reaction scope was explored by changing both the α-bromoketone and hydrazine hydrochloride portions, resulting in a series of complex drug-like small molecules in high yield (Scheme 8).

**Scheme 9:**
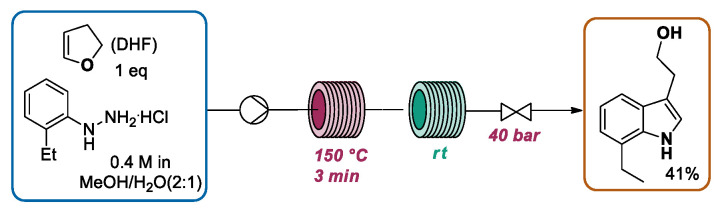
CF synthesis of 7-ET using conventional heating.

A very detailed study on the preparation of 7-ethyltryptophol (7-ET) **14** was proposed also by Kappe’s group [52]. Starting from previous results reported by Su and co-workers, concerning the kilogram scale synthesis of **14** using a flow system consisting of a three-pump and two-reactor flow system [53], Kappe proposed a new, operationally simpler, single pump one-reactor setup for this transformation. Additionally, in this work a precise study of the reaction mechanism was conducted in order to reduce the formation of several byproducts. In particular, a premixed solution of the hydrazine hydrochloride and DHF (dihydrofuran) in MeOH/H_2_O (2:1) was pumped using a syringe pump through a system composed of a Hastelloy loop immersed in an oil bath (150 °C), a short stainless steel loop for reaction cooling, and a back-pressure regulator (40 bar). By extraction with MTBE/PE, the majority of byproducts can be removed, obtaining 7-ET in a 41% yield (after column chromatography) with only 3 min of residence time (Scheme 9).

**Scheme 10:**
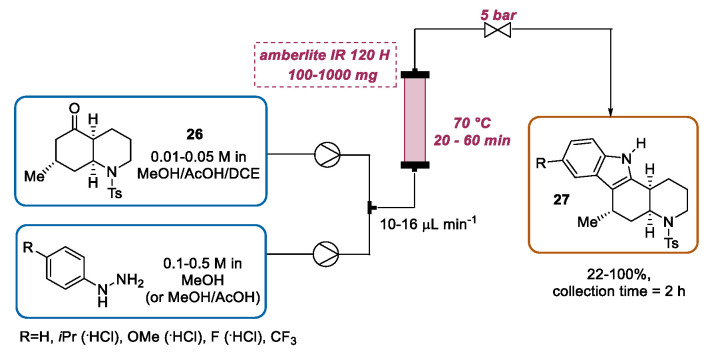
Amberlite IR 120 H-catalyzed continuous flow Fischer indole synthesis of pyrido[2,3-*a*]carbazoles.

Inspired by Watts’s work, recent articles by Bonjoch and Rutjes emphasized the use of heterogeneous catalysis using the polymeric sulfonic acid resin (Amberlite^®^ IR 120 H) for the continuous flow Fischer indole synthesis of pyrido [2,3-*a*]carbazoles [54]. For the initial experiments, methanol solutions of cyclohexanone **9** and phenylhydrazine **10**, selected as model substrates, were separately driven by syringe pumps through a flow system composed of a T-mixer unit, an ETFE cartridge packed with 100 mg of Amberlite^®^ IR 120 H (placed in oil bath), and a back-pressure regulator. Using a residence time of 10 min or 5 min, respectively, at 50 °C or 90 °C, the full conversion of the starting materials was observed. Additionally, the resin can be reactivated with a 10% H_2_SO_4_ solution, when a reduction in efficiency was detected. Similarly, a series of pyrido [2,3-*a*]carbazoles were prepared by the reaction of 5-oxo-*cis*-decahydroquinolines with a range of phenylhydrazines. However, adapting this setup for the preparation of pyrido[2,3-*a*]carbazoles, some slight modifications are needed. Firstly, the adding of AcOH as a cosolvent was necessary to avoid a side reaction mediated by the ammonia liberated during the indolization reaction. In order to avoid solubility problems, in some cases a ternary solvent mixture MeOH-AcOH-DCE (in different proportions) was used. Moreover, the reaction temperature was set at 70 °C and the residence time was tuned for each phenylhydrazine (or its hydrochloride salt) in a range between 20 and 60 min, affording different indole derivatives **27** as single diastereoisomers in satisfactory yields. (Scheme 10).

**Scheme 11:**
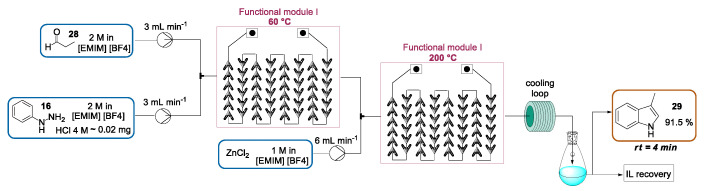
A continuous-flow Fischer indole synthesis of 3-methylindole in an ionic liquid.

Recently, Xu and Lv presented the Fischer indole preparation of 3-methylindole **29** in an ionic liquid—namely, 1-ethyl-3-methylimidazolium tetrafluoroborate ([EMIM] [BF4])—in the presence of ZnCl_2_ as a stoichiometric Lewis acid catalyst [55]. As stated above, also in this work a study on the decomposition of the hydrazone intermediate under batch conditions confirmed that the [3,3]-sigmatropic rearrangement was favored at high temperatures. However, the use of high temperatures and long reaction times also led to an increase in side-reactions. In this contribution, the authors showed how the excellent heat and mass transfer exhibited in flow microreactors enabled a high-yield preparation of 3-methylindole, with a concomitant decrease in the reaction time and control of by-product formation. According to optimized conditions, a solution of aldehyde **28** and a solution of phenylhydrazine, introduced by two syringe pumps, were mixed and the resulting solution passed into a Corning G1 reactor (functional module I) heated to 60 °C and generating hydrazone. The latter was mixed with a solution of ZnCl_2_ (60 °C). Subsequently, the mixture was directed through a Corning G1 reactor (functional module II) heated to 200 °C for the indole ring formation. Then, the mixture was cooled into a residence loop, collected, diluted with water, and extracted with toluene. By using a residence time of 4 min, 3-methylindole **29** could be prepared in a a 95% yield with a purity of 96% (net isolated yield of 91%) and eventually further purified by column chromatography or recrystallization. By the extraction of the aqueous phase with dichloromethane, most ionic liquid (85–86%) could be recovered and reused three times in the same transformation without any relevant decrease in the net isolated yield (Scheme 11).

### 2.2. Hemetsberger-Knittel Indole Synthesis under Continuous Flow Conditions

**Scheme 12:**
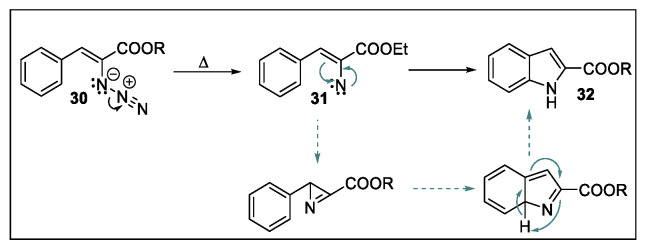
Hemetsberger-Knittel reaction.

The Hemetsberger-Knittel reaction, presented for the first time in 1969 [56], concerns the thermolysis of α-azidocinnamate esters **30** to give indole-2-carboxylic esters **32** [57]. The precise mechanism of this transformation is still unclear, but it is likely that the thermolysis of **30** leads to the formation of a highly electrophilic singlet nitrene intermediate species **31** [58], which formally undergoes a C-H insertion, giving the desired indole nucleus (Scheme 12). Moreover, 2H-azirines, deriving from the insertion of **31** to the adjacent double bond, have been also proposed as the reaction intermediates [59].

**Scheme 13:**
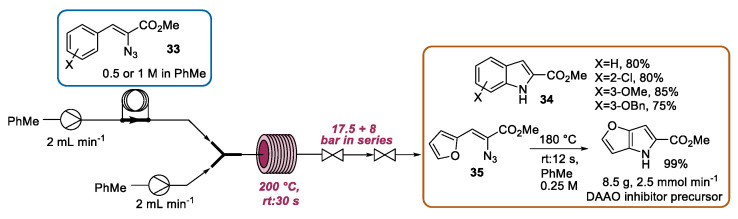
Hemetsberger–Knittel synthesis under continuous flow conditions.

The continuous flow preparation of a series of indoles based on the Hemetsberger–Knittel reaction was presented by Seeberger [60]. A solution of the azidoacrylate **33** in toluene was loaded into the reactor via an injection loop. The concentration (1.0 or 0.5 M) of the reactant solution was halved by mixing it with the second pump, which runs at the same flow rate. Then, the resulting solution passed through a 2 mL stainless steel heating loop (220 °C). By using these conditions, the high-yielding synthesis of indoles **34** was achieved with a residence time of 30 s (Scheme 13). This flow approach was able to address the safety issues related to the employment of azides; the use of high-boiling solvents, such as mesitylene and xylenes; and the scalability problems encountered under traditional batch conditions. In this context, the authors carried out a scalable continuous flow thermolysis of azidoacrylates with the preparation of 8.5 g of **35**, a precursor of a DAAO inhibitor, within 21 min (productivity of 2.5 mmol·min^−1^).

**Scheme 14:**
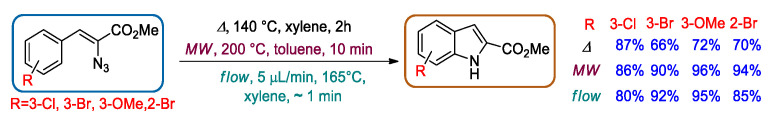
Preparation of a series of indoles by Hemetsberger–Knittel synthesis under different conditions.

In 2013, a comparative study between thermolysis, microwave irradiation, and flow approach for the preparation of indole-2-carboxylates via the Hemetsberger–Knittel reaction was presented by Jones [61]. By using microwave irradiation, indole-2-carboxylates were obtained in a higher yield and with a shorter reaction time (200 °C, 10 min) with respect to batch (140 °C, 2 h). By using the flow system, not only were similar reaction yields observed in comparison with the microwave approach, but also a further decrease in the reaction time (165 °C, ca. 1 min) was achieved (Scheme 14).

### 2.3. Indolization by Reductive Cyclization of Nitro Compounds

The reductive cyclization of aromatic nitro compounds is one of the most useful approaches for indole ring construction. The reduction step is generally performed by catalytic hydrogenation over Pd/C, Pt/C, or a combination of Raney nickel and hydrazine [62]. However, other reducing agents such as sodium dithionite, nickel boride/hydrazine, iron or zinc in acetic acid, stannous chloride, TiCl_2_-HCl, and TiCl_3_ have also been employed [16]. In this section, a classification of reductive cyclizations based on different starting aromatic nitro compounds has been proposed. In particular, the reductive cyclization of *o*-nitrobenzylcarbonyls, dinitrostyrenes, *o*-nitrophenylacetonitriles, *o*-nitrostyrenes, and β-(dimethylamino)-2-nitrostyrenes (generated from *o*-nitrotoluene in the Leimgruber–Batcho indole synthesis) has been described. In recent years, the intensification of some of these transformations under continuous flow conditions has been proposed, and this will be the topic of this section.

**Scheme 15:**
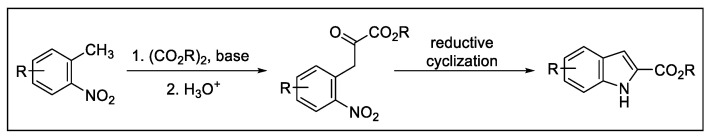
Reissert indole synthesis.

In this context, the Reissert indole synthesis is the classical method for the reductive cyclization of *o*-nitrobenzylcarbonyl compounds. In this reaction, *o*-nitrotoluene was reacted with an oxalic ester to give an *o*-nitrophenylpyruvate derivative which undergoes reductive cyclization, furnishing an indole-2-carboxylic acid derivative (Scheme 15).

**Scheme 16:**
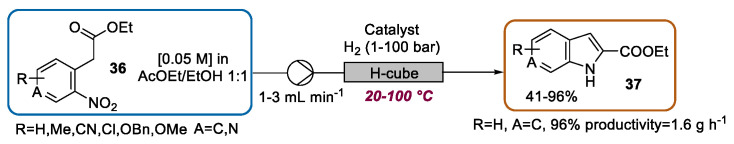
Intensification of the second step of Reissert indole synthesis under CF.

In 2011, Guillier and coworkers proposed a study focused on the second step of the Reissert indole synthesis. The authors reported a continuous flow preparation of various indole-2-carboxylic acid ethyl esters **37** by the reduction of the nitro group of **36**, followed by spontaneous cyclization (Scheme 16) [63]. For this purpose, they used the H-cube system, which ensured the preparation of indoles **37** in an extremely short time when compared to classical batch Pd/C catalyzed hydrogenation [64]. Moreover, with respect to other reduction procedures, such as Zn/AcOH [65] and microwave-assisted hydrogenation with cyclohexene [66], the presented flow approach was able to prevent tedious filtration and extraction steps, which may be detrimental for large-scale production. Once the advantages of the flow approach were established, a series of indoles were prepared by finely selecting the appropriate reaction conditions in terms of catalyst (Pd/C, Ru/C, Raney Ni), temperature (20–100 °C), pressure (1–100 bar), cartridge dimension (s-cart or l-cart), and flow rate (1–3 mL·min^−1^), depending on the reaction substrate. Furthermore, by means of a design-of-experiments (DoE) approach using the Design-Expert^®^ software [67], a precise process scale-up based on the evaluation of five parameters (temperature, pressure, catalyst type, solvent, and the presence of AcOH) was conducted. In particular, employing optimized conditions (3 mL·min^−1^, 0.05 M, 50 °C, 100 bars, EtOH/EtOAc 1:1, the absence of AcOH, Pd/C 1-cart), 11.1 g of one indole were produced within 6.75 h (1.6 g·h^−1^).

**Scheme 17:**
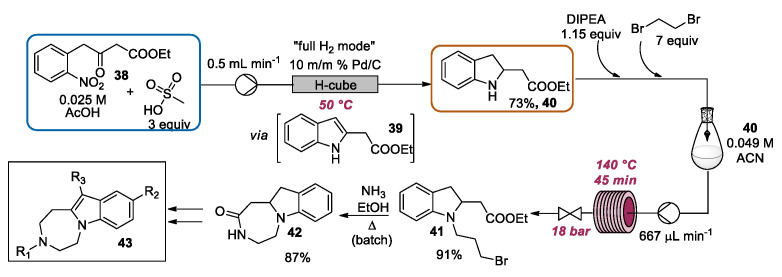
CF reductive cyclization of *o*-nitrobenzylcarbonyl en route to indole derivatives of general formula 43.

In the context of the reductive cyclization of *o*-nitrobenzylcarbonyl compounds, Örkényi and coworkers described the advantages of the continuous flow approach for the preparation of **40** (Scheme 17), an intermediate en route to 3,9,11-trisubstituted-1*H*,2*H*,3*H*,4*H*,5*H*-[1,4]diazepino[1,7-a]indole derivatives of general formula **43**, which are melanin-concentrating hormone receptor 1 (MCHr1) antagonists [68]. Under traditional batch conditions, 2-(2,3-dihydro-1*H*-indol-2-yl) acetate **40** can be synthesized by a two-step procedure, starting from 4-(2-nitrophenyl)-3-oxobutanoate **38**. The nitro-group reduction and cyclization afforded the indole **39**, which can be further reduced to give the indoline derivative **40**. In this work, a consecutive catalytic hydrogenation method to obtain **40** in one single-step from ethyl **38**, and without the isolation of indole **39**, was implemented. According to the optimized conditions, a solution of **38**, methanesulfonic acid, and acetic acid was pumped through a H-cube system with a 10 mol% Pd/C catalyst cartridge. By using a temperature of 50 °C and a flow rate of 0.5 mL·min^−1^, the full conversion of 4-(2-nitrophenyl)-3-oxobutanoate was observed, with a selectivity towards 2-(2,3-dihydro-1H-indol-2-yl) **40** of 73% determined by LC-DAD-MS. Only 3% of indole **39** was detected, whereas the remaining part was composed of side-products (12%). In the same contribution, also the following *N*-alkylation step for the preparation of **41** was intensified, exploiting flow chemistry tools. Under traditional batch mode, the *N*-alkylation reaction of **40** to afford the adduct **41** required a high excess (20–60 equiv.) of the carcinogenic 1,2-dibromoethane and used the high dilution technique to prevent the formation of by-products. Moreover, a long reaction time (up to 4 days) is needed. According to optimized conditions, by the DOE approach, a solution of **40**, DIPEA, and 1,2-dibromoethane in CH_3_CN was directed through a home-built 30 mL tube reactor heated at 140 °C. The system pressure was controlled using 18 bar back-pressure regulator. In this way, **41** was obtained in a high yield in less than 30 min, with a productivity ca. 200 times better as compared to the slow batch protocol. Moreover, the amounts of 1,2-dibromoethane can be significantly reduced to seven equivalents. Finally, due to the solubility issues of the product, the ring closure step mediated by ammonia was performed under batch conditions, affording the key intermediate 1*H*,2*H*,3*H*,4*H*,5*H*,11*H*,11a*H*-[1,4]diazepino[1,7-*a*]indol-2-one **42** in high yield.

**Scheme 18:**
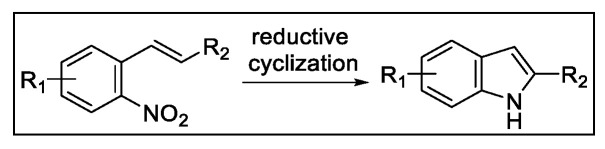
Reductive cyclization of *o*-nitrostyrenes.

In the 1960s, the preparation of indoles by the reductive cyclization of *o*-nitrostyrenes was reported by Cadogan [69,70] and Sundberg [71,72] (Scheme 18). This deoxygenative cyclization was typically performed by heating the aromatic nitro compounds in the presence of boiling trivalent phosphorus compounds, such as triethyl phosphite. However, this methodology suffered from the use of extreme conditions along with the generation of large quantities of phosphorous waste. Later, transition metal-catalyzed versions of this reaction using carbon monoxide (CO) as terminal reductant were proposed as suitable methodologies for indole ring construction [73,74,75,76,77,78,79,80,81,82,83,84]. Despite its synthetic utility, this multiphase deoxygenation is limited by the employment of highly toxic CO under elevated pressure and temperature [16].

**Scheme 19:**
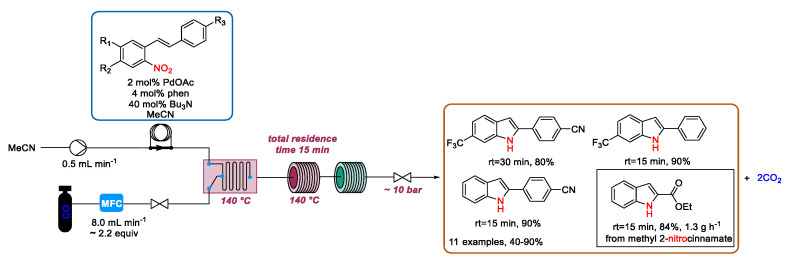
CF reductive cyclization of *o*-vinylnitrobenzenes using CO as a stoichiometric reductant.

In this regard, in 2017 Kappe and Roberge reported a continuous flow preparation of various 2-aryl-substituted indoles by the palladium-catalyzed reductive cyclization of *o*-vinylnitrobenzenes (*o*-nitrostilbenes and -styrenes), employing carbon monoxide as the stoichiometric reducing agent [85] (Scheme 19). Due to the larger specific interfacial area with respect to batch reactors, the availability of mass flow controllers to finely dose the gases following stoichiometry, and the possibility to improve the solubility of the gas in the liquid phase, adopting pressurized systems, continuous flow microreactors emerge as an enabling technology to boost this type of gas-liquid transformations. By using optimized conditions, a solution of nitrostilbene, Pd(OAc)_2_, and 1,10-phenanthroline/tributylamine in MeCN was loaded into an injection loop and introduced into the reactor at a 0.5 mL·min^−1^ flow rate. The mixture was combined in a glass static mixer at 140 °C with the CO stream (8 mL·min^−1^), which was directed from a gas cylinder to the system by using a mass flow controller. By using a fluoropolymer tubing, the mixer was connected with the residence coil reactor heated at 140 °C. Finally, the solution passed through a short cooling loop and a back-pressure regulator. The total residence time was 15 min, though some reaction output was reprocessed (total residence time of 30 min) using the same reaction conditions to improve the conversion. Immediately after the contact with CO, palladium (II) acetate was reduced to palladium (0), which partly deposited inside the channels of the flow device. According to the authors, this irreversible event induced the stopping of the reaction, explaining the low conversion observed in the case of less reactive nitrostilbenes. However, using this approach various 2-aryl-substituted indoles were prepared within 15 to 30 min, which is a significantly reduced reaction time with respect to the reported batch processes. In many cases, by HPLC-UV/Vis analysis the desired indole was identified as the only product in the crude mixture and therefore was easily isolated in high purity without needing column chromatography. Furthermore, the reaction was scaled up by employing a slightly different setup, in which the injection loop was removed and a high-pressure syringe pump were used instead of the HPLC pumps. Hence, by using methyl 2-nitrocinnamate as a starting material and 1 mol% catalyst loading, the corresponding indole was obtained in an 84% isolated yield with a throughput of 1.3 g·h^−1^ (Scheme 19).

**Scheme 20:**
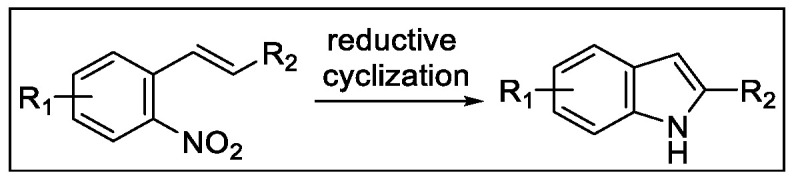
Reductive cyclization of *o*-nitrophenylacetonitriles.

As mentioned in the introduction of this section, *o*-nitrophenylacetonitriles have also been successfully employed for the preparation of the indole ring by reductive cyclization, in particular accessing 2,3-unsubstituted and 3-substituted indoles (Scheme 20).

**Scheme 21:**
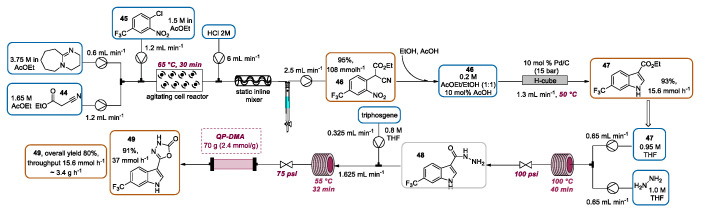
CF synthesis of indole-3-carboxylic ester by reductive cyclization and its derivatization to an auxin mimic.

Baxendale and coworkers presented the continuous flow preparation of indole-3-carboxylic ester **47** by the Pd-catalyzed reductive cyclization of *o*-nitrophenylacetonitrile **46** [86]. The indole **47** was then employed for the production of a novel mimic-based herbicide **49**. Firstly, for the synthesis of *o*-nitrophenylacetonitrile **46**, a base-mediated S_N_Ar was performed. A solution of DBU (base) and a solution of ethyl cyanoacetate **44** were mixed and then combined with a solution of 2-chloronitrobenzene **45**. The resulting mixture was directed into the Coflore ACR device, which was agitated at 8 Hz and maintained at the temperature of 65 °C to give adduct 46. The combined stream was quenched with a 2 M hydrochloric using an inline static mixing element, and the biphasic mixture was collected. EtOAc was selected as the best solvent for the first step, due to its compatibility with the following Pd-catalyzed reductive cyclization. Hence, after the dilution with EtOH and the adding of AcOH (10 mol%), the organic phase was drawn and pumped through a 10 mol% Pd/C catalyst cartridge placed into an H-cube. The cartridge was heated to 50 °C and pressurized at 15 bar, giving the desired indole **47** with a productivity of 3.7 g·h^−1^. Next, **47** was purified and functionalized by a telescoped two-step sequence (Scheme 21). In particular, the substitution of the ethoxy group by hydrazine was achieved by mixing a THF solution of **47** with a THF solution of hydrazine. The resulting solution was passed through a FEP flow coil heated to 100 °C, using a back-pressure regulator of 100 psi to control the system pressure. The resulting acyl hydrazine **48** was directly reacted with a solution of CDI in a FEP flow coil (heated to 75 °C) giving, after passing through a scavenging cartridge of QP-SA (a sulfonic acid functionalized polymer), the desired 3H-[1,3,4]oxadiazol-2-one **49** in an 82% yield. Alternatively, the latter cyclization can be achieved by combining acyl hydrazine **48** with an input of triphosgene (in CHCl_3_ solution) and directing it through a packed bed scavenging cartridge containing QP-DMA (*N*,*N*-dimethylbenzylamine polystyrene), obtaining, after solvent evaporation, the desired final product 49 in a 91% yield, without the need for further purification. 

### 2.4. Heumann Indole Synthesis

**Scheme 22:**
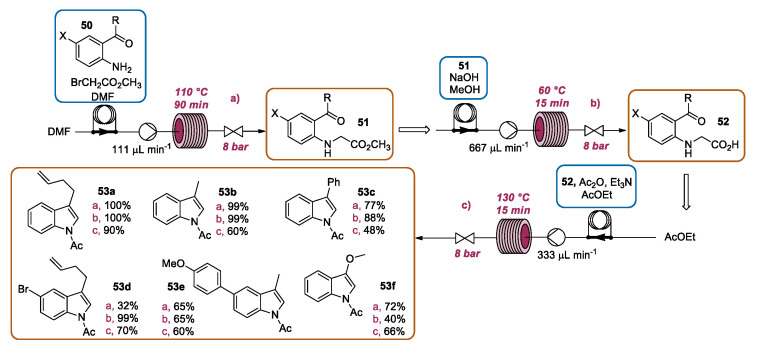
CF intensification of three-step Heumann indole synthesis: (**a**) alkylation of aniline **50**; (**b**) saponification; (**c**) Heumann cyclization.

Very recently, Lubell and coworkers reported the continuous flow intensification of the Heumann indole synthesis. This transformation was initially proposed for the preparation of indigo, a relevant dyestuff, although a different protocol was selected for the industrial preparation of this molecule, the so-called Heumann−Pfleger method [87]. Almost forgotten, in the following decades, the Heumann indole synthesis has been sporadically utilized for the preparation of substituted indoles [88,89,90,91,92,93,94]. In this paper, a useful reaction was successfully exploited for the preparation of a series of 3-substituted indoles by three consecutive flow steps. The sequence was optimized for the preparation of 3-(but-3-enyl)indole **53a** (Scheme 22a). In the first step, methyl bromoacetate and 1-[2-aminophenyl]pent-4-en-1-one **50** were premixed in DMF and loaded into an injection loop. By using DMF as the elution solvent, the mixture was directed into a PFA reactor heated to 110 °C. After a residence time of 90 min, the N-alkylated intermediate methyl [2-(pent-4-enoyl)phenyl]glycinate **51** was obtained in a quantitative yield, which was a remarkable result when compared to the best result obtained under batch conditions (57%). The subsequent saponification could be effectively achieved using both the batch and flow approaches. The latter was performed by loading a methanol mixture of **51** and aqueous NaOH solution (10 N) into an injection loop. MeOH was used as a solvent to flow the mixture into a PFA reactor heated to 60 °C with a residence time of 15 min, obtaining the 2-[(pent-4-enoyl)phenyl)glycine **52** in a quantitative yield (Scheme 22b). The latter was mixed with acetic anhydride and Et_3_N in AcOEt and the mixture was injected into an injection loop. The same solvent was used to introduce the mixture through a stainless steel reactor, allowing the Heumann cyclization for the preparation of desired 1-acetyl-3-(but-3-enyl)indole **53** in a 90% yield (Scheme 22c), which was also in this case higher with respect to the batch reaction (74%). The same protocol was extended for the preparation of different 3-substituted indoles **53b**–**f**. Moreover, by using this flow protocol, the indoles were synthesized in a high purity without needing chromatography purification.

### 2.5. Indole Synthesis through Photochemical Benzannulation

**Scheme 23:**
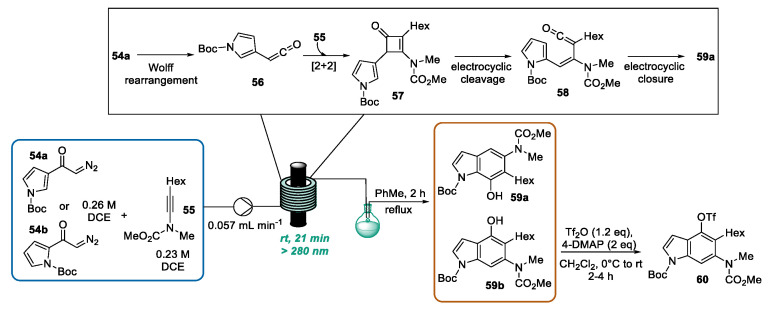
CF synthesis of highly substituted indoles by photochemical benzanullation.

It is well established that the advent of flow chemistry enables researchers to face the longstanding restrictions of batch photochemistry, such as limited scale-up, inefficient light transmission, and prolonged reaction times [95,96]. Flow microreactors are typically characterized by a high surface-to-volume ratio, which permits a more uniform irradiation of the entire reaction mixture, often allowing a decrease in photocatalyst loadings, along with a considerable reduction in the reaction time from hours/days in batch to seconds/mins in flow. The latter is not only convenient for the process productivity but is also important in preventing side-reactions. In 2013, Danheiser proposed a flow photochemical benzannulation for the preparation of various aromatic and heteroaromatic compounds [97]. Among them, two highly functionalized indole derivatives were also obtained. By using a continuous flow photochemical reactor, a solution of diazoketone of type **54a**–**b** and ynamide **55** in DCE was pumped through the system by a syringe pump (Scheme 23). The light-promoted Wolff rearrangement of diazo ketone furnished vinylketene **56**. According to the proposed “pericyclic cascade” mechanism, vinylketene **56** reacted with ynamides **55**, leading to vinylcyclobutenone **57**. The latter undergoes an electrocyclic cleavage triggered by light, giving dienylketene **58**, which furnished indole derivative **59** after a rapid 6-π electrocyclic ring-closure and tautomerization. Due to the uncomplete conversion of vinylcyclobutenone **57** into the desired indole derivative **59**, the reaction mixture was collected in a pear-shaped flask, concentrated, dissolved in toluene, and refluxed for 2 h. Due to its air sensitivity, indole **59b** was converted into the relative triflate adduct **60** to facilitate the purification step.

## 3. Derivatization of Indole Ring under Continuous Flow Conditions

In recent years, flow chemistry has been applied not only for the construction of the indole nucleus but also for its efficient derivatization. Hence, in the following sections, the attention will be focused on the efforts made by different groups in the intensification of indole derivatization procedures using flow approach emphasizing the advantages over traditional batch chemistry.

### 3.1. Indole Ring Derivatization Mediated by Visible Light-Photoredox Catalysis in Flow Mode

**Scheme 24:**
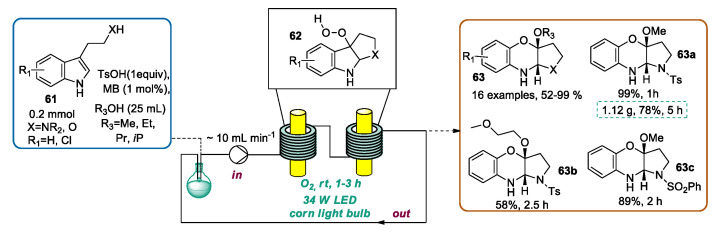
CF preparation of tricyclo-1,4-benzoxazines library via metal-free visible-light photoredox catalysis.

As briefly described in Section 2.5, flow chemistry offers some technological features that arise as extremely useful in view of an intensification of photochemical processes. In the field of indole photochemical derivatization, Chen et al. reported a continuous flow preparation of tricyclo-1,4-benzoxazines by the intramolecular cyclization of indole derivatives via visible-light photoredox catalysis [98]. For this study, a homemade capillary photoreactor consisting of two quartz glass tubes connected in series, described and named by the authors as “reaction towers”, was used. A fluorinated ethylene propylene FEP tube was wrapped around each reaction tower which held a LED corn light bulb (34 W) in the center, thus providing the reaction mixture irradiation [99]. According to the optimized protocol, indole derivative **61**, *p*-toluensulfonic acid (TsOH), a catalytic amount of methylene blue (MB), and the selected alcohol were mixed in a round bottom flask. The resulting mixture was sucked into the reactor at one end and returned at the other (rpm = 50, flow rate is about 10 mL·min^−1^). The transformation was performed under O_2_, giving the hydroperoxide intermediate **62** which, in the presence of acid (TsOH), provided the desired tricyclo-1,4-benzoxazine **63**. A series of tricyclo-1,4-benzoxazines were obtained by varying the starting indole derivative **61** (*N*-monosubstituted tryptamines or tryptophol were used) and the alcohol (methanol, ethanol, propanol, and isopropanol). Moreover, carrying out the transformation in CH_3_CN and adopting 2-methoxyethan-1-ol as the nucleophile, a moderate yield of the corresponding tricyclo-1,4-benzoxazine **63b** was observed. It is noteworthy that the transformation is complete in 1–3 h rather than the 24 h required by the batch protocol. Furthermore, a gram-scale experiment was performed, obtaining the desired product **63a** in a 78% yield in 5 h (Scheme 24).

**Scheme 25:**
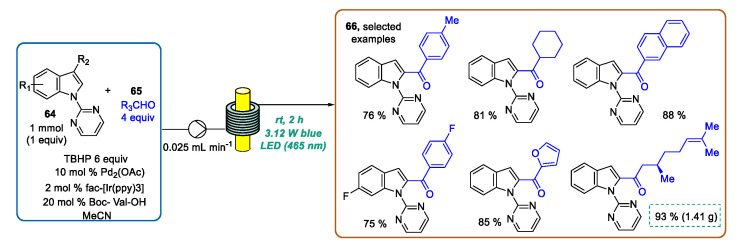
CF C-2 acylation of indoles via dual photoredox/palladium catalysis at room temperature.

A further proof of the benign effect of the homogenous irradiation ensured by continuous photoflow synthesis has been furnished by Noël, Van der Eycken, and coworkers [100]. These authors reported the C-2 acylation of *N*-pyrimidylindoles **64** using aldehydes as acyl surrogates by merging visible-light photoredox catalysis and palladium catalyzed C-H activation. The pyrimidyl ring linked to the indole nitrogen acted as a directing group assisting the C-2 functionalization, and could be removed at the end of the sequence. The transformation was performed at room temperature under batch and flow conditions. Regarding the flow approach, a 3 mL PFA capillary reactor was wrapped around the inner cylinder of a 3D-printed photomicroreactor in combination with a 3.12 W blue LED (λmax = 465 nm) light source. Using a syringe pump, a solution of **64**, Pd(OAc)_2_, fac-[Ir(ppy)_3_] as a photocatalyst, Boc-protected l-valine (Boc-Val-OH), aldehyde **65**, and tert-butyl hydroperoxide (TBHP) as the oxidant in anhydrous acetonitrile were introduced into the photomicroreactor at a flow rate of 0.025 mL·min^−1^, corresponding to a residence time of 2 h. Aromatic and aliphatic aldehydes were successfully employed as acylating partners, obtaining the functionalized indoles **66** in good to excellent yields. Moreover, the reaction scope could be extended by using a series of differently substituted *N*-pyrimidylindoles **64**. By a systematic comparison with the traditional batch setup, the authors demonstrated that the designed flow approach enabled the acceleration of the reaction time from 20 h to 2 h, the reduction in catalyst loading from 2 mol% to 0.5 mol%, and a general increase in isolated yields. In addition, by using a numbering-up approach (2 × 3 mL), the continuous flow C-2 acylation of *N*-pyrimidylindole **64** using (−)-citronellal as a coupling partner was accomplished, obtaining 1.41 g (93%) of the desired product within less than 9 h (Scheme 25).

### 3.2. Continuous Flow Functionalization of Indole Nitrogen

**Scheme 26:**
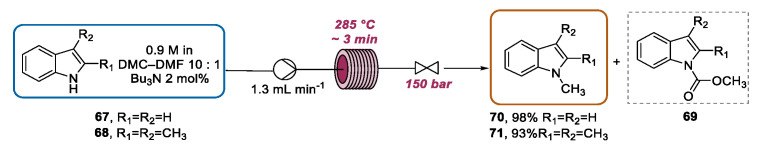
CF *N*-methylation of indole derivatives.

As described in Section 2.2, the attractive possibility of translating efficient microwave batch protocols into high-temperature/high-pressure scalable continuous flow process was extensively investigated. This kind of approach has been applied to the functionalization of the indole ring by Kappe and Holbrey. These authors reported an efficient *N*-methylation of indole **67** with dimethylcarbonate (DMC), using tributylmethylammonium methylcarbonate, an ionic liquid, as the catalytic base [101]. Initially, this transformation was carefully optimized under microwave batch mode, finding that, at reaction temperatures below 150 °C, the major reaction product was *N*-(methoxycarbonyl)indole **69**, and the desired *N*-methylindole **70** could not be detected. In order to selectively drive the transformation toward the formation of **70**, the use of a temperature of 230 °C for 20 min at a pressure of ~17 bar was necessary. Moreover, given that tributylmethylammonium methylcarbonate is prepared by the treatment of tributylamine with DMC [102], the authors successfully proposed to generate the catalyst in situ by adding tributylamine to a solution of indole **67** in DMC–DMF (10:1, *v*/*v*) and subjecting the mixture to microwave heating. Once the optimized conditions under batch microwave mode were established, the transformation was transferred into a high-temperature/high-pressure continuous flow reactor (X-Cube Flash) containing a stainless steel coil, a heat exchanger, and a system pressure valve to set the pressure value. Firstly, by using a 16 mL stainless steel coil and a flow rate of 1.2 mL·min^−1^, the optimized microwave batch conditions (230 °C, 20 min) were translated to the flow apparatus, obtaining the total conversion of indole **67** into **70**. Subsequently, the process was further intensified by pumping the mixture of **67** and tributylamine in DMC-DMF (10:1) with a flow rate of 1.3 mL·min^−1^ (~3 min of residence time) through a 4 mL stainless steel coil heated to 285 °C and with the pressure set to 150 bar. The methylation protocol was extended to indole **68** derivative and amines, phenols, thiophenols, and carboxylic acids. In all the cases, the full conversion of the starting material was observed. Using this protocol, 250–370 mg of product could be obtained within 3 min, which corresponded to a calculated throughput of ~100 g per day (Scheme 26).

### 3.3. Continuous Flow Functionalization of C-3 Position of Indole Ring

**Scheme 27:**
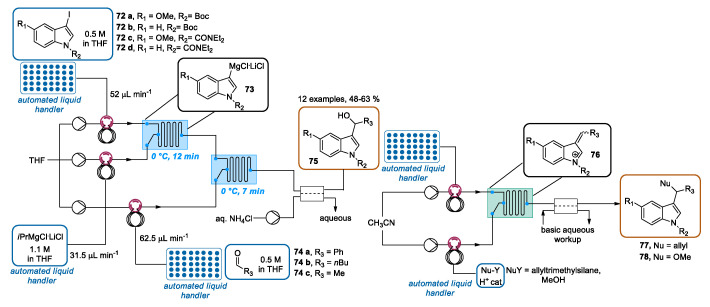
CF automated multi-step generation of 3-hydroxymethylindoles and their conversion to **77** and **78** via acid-catalyzed nucleophilic substitution.

In recent years, different groups have reported the decoration of the indole ring at the C-3 position by using continuous flow approaches. In this context, O’Shea designed an inspiring automated sequential approach for the construction of a 3-indolylmethyl derivatives library [103]. Firstly, 12 3-hydroxymethylindoles were prepared by the iodo–magnesium exchange of four 3-iodoindoles, followed by trapping with three different aldehydes. The halogen-magnesium exchange reaction was performed by introducing indole solutions **72** and iPrMgCl·LiCl with automated injection loops into a reactor chip at 0 °C with a residence time of 12 min. The output flow containing the 3-metallated indoles **73** was reacted with solutions of aldehydes **74** in a second reactor chip, using a residence time of 7 min at 0 °C. Then, the resulting mixture was combined with an aqueous solution of NH_4_Cl in a liquid–liquid extraction chip. The collection of the organic phase furnished, after purification, the desired 3-hydroxymethylindoles **75**. It is noteworthy that this system setup was able to perform the 12 metalations, electrophile additions, in-line extractions, and product collections automatically. The entire system was controlled by a computer software program to judiciously calibrate the sequential reagents injections, extraction, and collection, and to set any other process parameter. Subsequently, exploiting again multi-microreactor setups in combination with inline flow liquid/liquid extractors, the obtained indoles were employed for the preparation of indole derivatives **77** and **78** through acid-catalyzed elimination–addition sequences via the intermediate electrophilic indolium cations **76**, using allyltrimethylsilane and methanol as nucleophiles. Hence, a collection of 36 indole derivatives was obtained starting from only four indoles by merging continuous multi-step systems and continuous-flow extraction technology (Scheme 27).

**Scheme 28:**
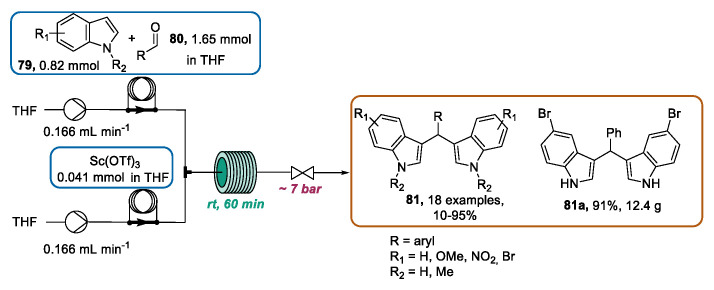
Sc(OTf)_3_ catalyzed continuous flow synthesis of BIM_S_ 81.

In 2017, Ley and coworkers furnished a contribution to the field, reporting the scandium triflate (Sc(OTf)_3_) catalyzed preparation of a series of bis(indolyl)methanes (BIM_S_) [104], which are biological relevant derivatives [105,106]. In this article, the batch protocol previously reported by Sato et al. [107] was translated to flow mode with the careful optimization of the reaction solvent, solution flow rates and concentrations, reactor volume, reaction time, and temperature. According to the best conditions, a solution of indole **79** and aldehyde **80** and a Sc(OTf)_3_ solution were mixed in a T-piece, and the resulting mixture was directed through a 20 mL reactor coil using a reaction time of 60 min, which is convenient with respect to the reported batch protocol. The designed flow approach, characterized by short reaction time and easy work-up operations, allowed the rapid exploration of the reaction scope. Hence, by using various indoles and aldehydes, a library of 18 BIM_S_ was obtained, including four novel structures, providing the opportunity to expand the biological utility of this class of molecules. Moreover, using a slightly different setup, the gram-scale preparation of bis(indolyl)methanes derivative **81a** was performed, obtaining 12.4 g with an operation time of 5 h and 46 min (Scheme 28).

**Scheme 29:**
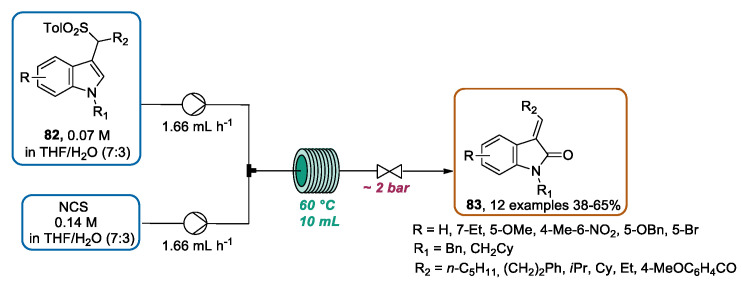
NCS-mediated oxidative conversion of sulfonyl indoles **82** into 3-alkylidene-2-oxindoles **83** under CF conditions.

A further contribution by Palmieri and coworkers presented the oxidative transformation of sulfonyl indoles **82** into 3-alkylidene-2-oxindoles **83** using the continuous flow approach [108]. The reaction involved indole ring oxidation mediated by *N*-chlorosuccinimide (NCS), followed by the elimination of arylsulfinic acid to introduce exocyclic unsaturation. Interestingly, the initial experiments under traditional batch conditions revealed only a low yield of the expected product **83** along with side-products probably derived from overoxidation pathways. Hence, in order to reduce the contact time between the obtained unsaturated oxindole **83** and the oxidizing reagent, a continuous flow approach was evaluated. A flow apparatus, consisting of two reservoirs, two HPLC pumps, a T-mixer, a PTFE coil reactor, and a back-pressure regulator, was designed for the optimization study. A careful evaluation of the solvent mixture, reactant concentrations, solution flow rates, and reaction temperature was conducted. Moreover, the authors showed how the use of a protecting-group on the indole nitrogen was essential for this transformation, identifying the benzyl group as the most suitable one. By using best conditions (Scheme 29), the sulfonyl indoles **82** and NCS solutions were directed from each reservoir to the reactor coil heated to 60 °C, obtaining various 3-alkylidene-2-oxindole derivatives in moderate to satisfactory yields. The obtained products possessed an E configuration, except for derivatives bearing a methyl group at the C-4 position of the indole ring, which were obtained as Z stereoisomers. Remarkably, this contribution represented the first approach for the direct conversion of 3-functionalized indole derivatives into 3-alkylidene-2-oxindoles, and it was achievable only under flow conditions. 

**Scheme 30:**
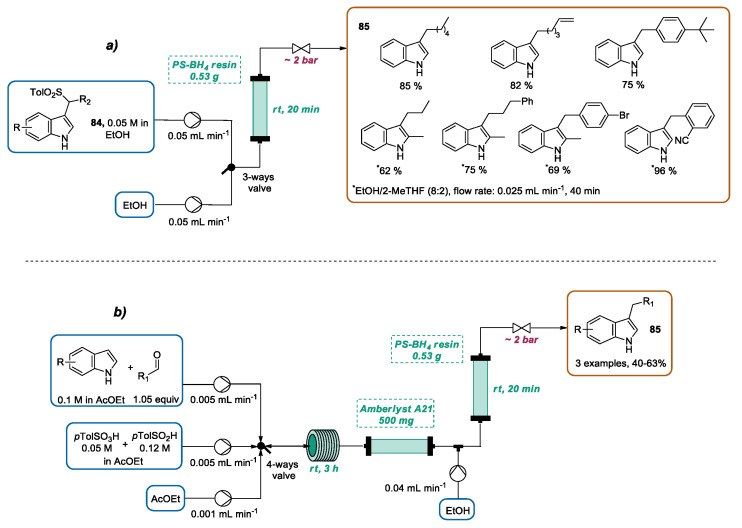
(**a**) CF preparation of 3-alkylated indoles by the reduction of sulfonyl indoles and (**b**) integrate flow setup for the synthesis of 3-alkylindoles directly from their remote precursor’ indoles and aldehydes.

Very recently, the same group presented the continuous flow preparation of 3-alkylindoles **85** through the polymer-supported sodium borohydride reduction of 3-(1-arylsulfonylalkyl) indoles **84** (sulfonyl indoles) [109]. The reaction stoichiometry, reactant concentrations, residence time, and solvent were judiciously optimized by using a flow system composed of two syringe pumps, a three-way valve, a packed bed reactor, and a back-pressure regulator. According to the optimized conditions, the transformation was performed by directing a 0.05 M ethanolic solution of sulfonyl indole **84** through a packed bed reactor containing six equivalents of PS-BH_4_ resin using a residence time of 20 min. Once the **84** infusion was finished, the three-way valve was switched, permitting the ethanol entrance, which pushed the residual sulfonyl indole into the system. By using these conditions, a collection of 3-alkylindoles **85** was obtained in good yields. 2-MeTHF was added as a cosolvent when solubility issues were encountered, although a doubling of reaction time (halving the flow rate) was required in these cases (Scheme 30a). The authors stated that previous methodologies adopted for sulfonyl indole reduction using sodium borohydride suffer from tedious work-up operations, with consequent solvent consumption and waste production. In this work, by merging the flow chemistry and the employment of solid supported species, the work-up was limited to solvent evaporation, reducing the operational time and thus increasing the productivity. Since sulfonyl indoles **84** can be prepared by a three-component coupling of indoles with aldehydes and arylsulfinic acids, the authors designed an integrated flow setup for the synthesis of sulfonyl indoles **84** and subsequent inline sodium borohydride reduction (Scheme 30b). In this way, the one-pot generation of three 3-alkylindoles **85** directly from their remote precursors, indoles, and aldehydes was achievable. For this purpose, the flow apparatus schematized in Scheme 30b was used. Sulfonyl indoles **84** were obtained by pumping a solution of indole and aldehyde and a solution of *p*TolSO_3_H and *p*TolSO_2_H into a reactor coil using a residence time of 3 h. Then, the resulting mixture was passed through a packed bed reactor containing Amberlyst A21 to remove acidic species. Finally, the passage through the packed bed reactor containing PS-BH_4_ resin afforded the desired 3-alkylindoles **85**.

**Scheme 31:**
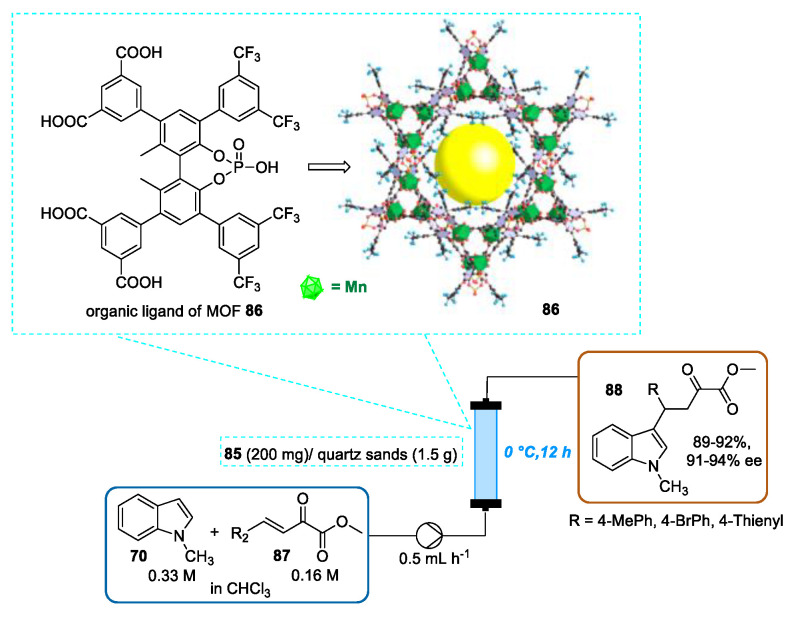
CF Friedel–Crafts alkylations of *N*-methylindole with β,γ-unsaturated α-ketoesters catalyzed by a metal-organic framework (copyright of Reference [7]).

In 2017, Liu and Cui reported the use of chiral metal−organic frameworks (MOFs) for the enantioselective Friedel-Crafts alkylation of indoles with a series of ketoesters under batch and flow conditions [110]. In this contribution, the authors demonstrated how the chemical stability, catalytic activity, and stereoselectivity of MOFs can be boosted by tuning the ligand structures. In particular, among three porous chiral MOFs with the framework formula [Mn_2_L(H_2_O)_2_], outstanding results were furnished by a porous chiral CF_3_-containing MOF **86**. By using a syringe pump, a solution of N-methylindole **70** and β,γ-unsaturated α-ketoesters **87** in CHCl_3_ was introduced into a stainless steel column filled with **86** (ground with a diameter of around 0.4–5 μm) and quartz sands (20–40 mesh) with a residence time of 12 h obtaining the Friedel-Crafts addition products **88** in 89–92% yield with 91–94% of enantioselectivity. With respect to batch experiments, a slight decrease in enantioselectivity probably related to the presence of silica used for the reactor packing was observed. However, under flow conditions, a slight increase in catalytic activity was achieved. The authors stated that the fixed-bed reactor can be reused up to seven times without any significant worsening of system performance. Notably, this work represented the first example of the use of MOF-mediated heterogeneous asymmetric catalysis under continuous flow conditions (Scheme 31).

**Scheme 32:**
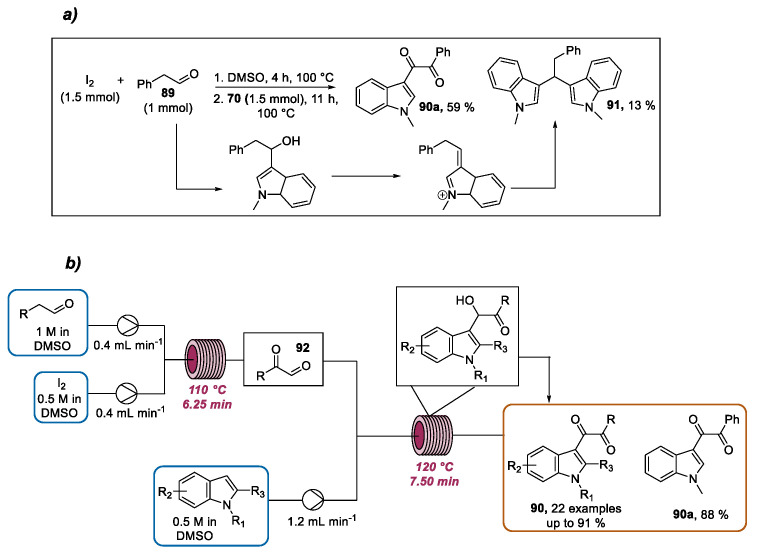
(**a**) Batch and (**b**) flow synthesis of dicarbonyl indoles via I_2_/DMSO-mediated oxidative coupling.

In 2018, the continuous flow preparation of C-3 dicarbonyl indoles via the I_2_/DMSO-mediated oxidative coupling of aryl acetaldehydes with indoles was proposed by Fang and Guo [111]. Initial batch experiments were conducted, using phenylacetaldehyde **89** and *N*-methylindole **70** as starting materials. According to the optimal batch conditions, a mixture of I_2_ (1.5 equivalents) and phenylacetaldehyde in DMSO was heated for 4 h. Then, *N*-methylindole was added and the solution was heated for 11 h, obtaining the dicarbonyl indole **90a** in a 59% yield (Scheme 32a). Despite the fact that *N*-methylindole **70** was added after phenylacetaldehyde **89**, the formation of by-product **91**, derived from dehydration between the acetaldehydes and indoles, was not negligible (13%). To face this limitation, a two-step continuous flow system was designed (Scheme 32b). In the first microreactor, the aryl acetaldehyde was subjected to Kornblum oxidation to produce the key intermediate phenyl glyoxal C **92**. In the second microreactor, **92** goes through a Friedel–Crafts-type reaction with indole, followed by an oxidation process to afford the desired dicarbonyl indole. The two reaction steps were separately optimized. According to the best conditions, a DMSO solution of **89** and a DMSO solution of I_2_ were introduced in the first reactor heated to 110 °C using a residence of time of 6.25 min, producing the phenyl glyoxal C intermediate. The latter was reacted with a DMSO solution of **70** in a second reactor heated to 120 °C with a residence time of 7.5 min, obtaining a high yield (88%) of **90a** with only traces of byproduct **91**. Therefore, the developed flow protocol ensured higher yields and a reduced reaction time and iodine dosage (0.5 equivalents), reducing the byproduct formation with respect to the batch setup. The reaction scope was then explored by varying both the aryl acetaldehydes and indole derivatives, producing a collection of dicarbonyl indoles. Notably, using this approach the use of a high iodine amount and the employment of amine catalyst, which are the main drawbacks of previously reported batch protocols, could be overcome.

### 3.4. Continuous Flow Functionalization of C-2 Position of Indole Ring

**Scheme 33:**
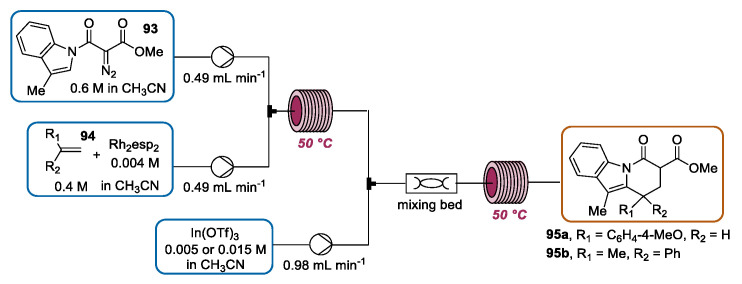
CF tandem, bicatalytic cyclopropanation-ring-opening cyclization.

As described above in this review, flow chemistry offers the possibility to access highly complex molecular structures through continuous flow multi-step strategies applicable for industrial purposes. In this context, Pollet and France reported a tandem, bicatalytic continuous flow cyclopropanation-homo-Nazarov-type ring-opening cyclization for the preparation of a hydropyrido[1,2-*a*]indole framework, which is present in various bioactive compounds [112]. Remarkably, this contribution was the first example in the literature of a homo-Nazarov-type cyclization under continuous flow conditions. The two steps of the sequence, cyclopropanation and ring-opening cyclization, were separately optimized in batch with great accuracy to develop an industrial-viable process in terms of solvent selection and catalyst loading in order to transfer the process in flow mode. An integrated, two-step setup was designed (Scheme 33). The first step, the Rh-catalyzed cyclopropanation, was performed by reacting a solution of diazoester **93** with a mixture of Rh_2_(esp)_2_ catalyst and styrene **94** in a stainless-steel reactor coil heated to 50 °C. The outcoming solution, characterized by an irregular volumetric flow rate due to the N_2_ generation, was mixed with a solution of In(OTf)_3_ in a mixing bed packed with 2 mm borosilicate glass beads. The resulting mixture was directed through a second stainless steel reactor coil heated to 50 °C to achieve the ring-opening cyclization. In the case of hydropyrido[1,2-*a*]indole **95a**, the continuous flow tandem approach ensured a higher yield when compared to the batch and an impressive throughput of 4.7 g·h^−1^. Otherwise, probably due to the flow irregularity observed during the cyclopropanation step and solubility issues, a lower yield was observed in the case of hydropyrido[1,2-*a*]indole **95b** in comparison to the batch result. However, a relevant throughput of 2.8 g·h^−1^ was achievable.

## 4. Conclusions

The relevance of indole, a biologically accepted pharmacophore in medicinal compounds, and its derivatives, requires a continuous improvement of their preparation. In this review article, we have tried to report the contribution of flow chemistry in the reinforcement of synthetic strategies for the indole nucleus construction and its functionalization, emphasizing the convenience of flow translation with respect to traditional batch procedures. We highlighted some aspects on the use of enabling flow technologies in the synthesis of indole cores, reporting representative examples using different microreactor devices, trying to show how the flow performance surpassed the traditional batch approach. The application of continuous flow chemistry for the fast generation of libraries of indole derivatives, useful for biological screening, is foreseen for the future. In addition, the incorporation of in-line biological assays of indole derivatives will speed up the process of drug discovery, placing continuous flow chemistry at the frontier of modern medicinal chemistry. Our perspective is that enabling technologies such as flow chemistry will become irreplaceable tools for modern chemical synthesis.
